# Smartphone adapters for flexible Nasolaryngoscopy: a systematic review

**DOI:** 10.1186/s40463-018-0279-6

**Published:** 2018-05-08

**Authors:** A. E. Quimby, S. Kohlert, L. Caulley, M. Bromwich

**Affiliations:** 1 Department of Otolaryngology - Head and Neck Surgery, University of Ottawa, 501 Smyth Rd, Module S, Room M2566, Box 216, Ottawa, ON K1H 8L6 Canada; 2 Bringham and Women’s Hospital, Department of Neuroendocrinology, 75 Francis St, Boston, MA 02115 USA; 3 Division of Otolaryngology – Head and Neck Surgery, Children’s Hospital of Eastern Ontario, 401 Smyth Rd, Ottawa, ON K1H 8L1 Canada

**Keywords:** Smartphone endoscope adapters, Flexible nasolaryngoscopy, Laryngoscopy, FNL

## Abstract

**Background:**

Flexible nasolaryngoscopy is an essential component of the otolaryngological physical exam. Historically, the ability to create and share video recordings of these endoscopic exams has been limited by poor mobility of fixed endoscopy towers. The advent of smartphone endoscope adapters has allowed physicians to create and share video recordings of endoscopy in a wide variety of locations that would not have previously been feasible. This paper sought to review the literature on the effect of smartphone endoscope adapters on patient care, patient satisfaction, and resident learning.

**Methods:**

This systematic review was conducted according to PRISMA guidelines. A systematic literature search was performed for all relevant English language studies (1946–2017) using Ovid MEDLINE, PubMed, and EMBASE. The study protocol was registered with the PROSPERO database.

**Results:**

A total of 91 abstracts were identified and screened by two independent reviewers. Based on inclusion and exclusion criteria, three studies were selected and subjected to full-text extraction as well as quality assessment. These studies demonstrated high diagnostic accuracy and quality of smartphone adapter-recorded videos, and a benefit of these devices on resident education. Due to the heterogeneity of included studies’ methods and measures, a meta-analysis was not possible, so a qualitative synthesis of the literature results was performed.

**Conclusion:**

Despite a paucity of data on the subject, the present study provided a comprehensive review of the literature, and suggested overall high diagnostic accuracy, quality, and enhancement of resident education with the use of smartphone endoscope adapters for flexible nasolaryngoscopy.

**Trial registration:**

CRD42018086714.

**Electronic supplementary material:**

The online version of this article (10.1186/s40463-018-0279-6) contains supplementary material, which is available to authorized users.

## Background

Flexible nasolaryngoscopy (FNL) provides invaluable insight to the practicing Otolaryngologist and can be used to diagnose a wide variety of pathology. It is an essential skill that must be mastered by all trainees in Otolaryngology - Head and Neck Surgery (OTOHNS).

While FNL is not technically challenging to the experienced otolaryngologist, there are many subtleties to its use and interpretation that may not be immediately appreciated by junior learners. As such, senior residents and attending physicians are often required to be present during the endoscopic exam to verify the junior trainee’s findings. As endoscopy towers are expensive and stationary, they are not conducive to remote locations such as emergency departments or patient wards, where patients commonly undergo FNL. Historically, FNL performed in these locations were not recorded and patients were then subjected to repeat examination, leading to redundancy and additional discomfort.

Smartphone endoscope adapters are a relatively new technology which provide a mechanism to record flexible nasolaryngoscopy examinations performed in remote locations using portable scopes. In doing so, they allow attending physicians to remotely provide opinion and advice following a single assessment performed with a portable scope. Recorded videos obtained in this manner may additionally be retained for educational or research purposes.

Since their introduction, there have been few articles examining the use of smartphone endoscope adapters. The purpose of this study was to perform a systematic review of the literature on the use of these devices. Specifically, we sought to assess the effect of smartphone endoscope adapters on video recording quality, patient satisfaction and care, and trainee educational experience. We also present our own institution’s experience with the use of smartphone endoscope adapters.

## Methods

A systematic review of the literature based on the Cochrane handbook and the Preferred Reporting Items for Systematic reviews and Meta-analyses (PRISMA) guidelines was performed [1, 2]. The study protocol was registered with the PROSPERO database (Trial Registration: CRD42018086714).

### Eligibility criteria

Studies were selected using Population, Intervention, Comparator, Outcome, Study Design (PICOS) guidelines. We included all studies from 1946 to September 2017 published in English language in peer-reviewed journals.

### Information sources and search strategy

With the help of an experienced librarian, we conducted a literature search using the following databases: Ovid MEDLINE, PubMed, and EMBASE. A MeSH terms and keywords search was conducted using truncation and adjacency operator and Boolean operators. MeSH terms were: endoscop*, otorhinolaryngologic diseases; otolaryngology; otorhinolaryngologic surgical procedures; nose; nasal; larynx*; laryngoscopy; laryngoscopes; nasolaryngoscop*; smart phone*; iphone*, andrid*, adaptor*, or adapter. We also performed a hand search of citations from relevant articles.

### Study selection

Study inclusion and exclusion criteria were clearly defined, and are illustrated in Table 1.

**Table 1:** Inclusion and Exclusion Criteria

Inclusion Criteria	
Population	○ Endoscopy performed by Otolaryngology residents/ physicians ○ Adult or pediatric patients
Intervention	○ Use of smartphone adapters for flexible nasolaryngoscopy
Comparator	○ Endoscopy tower video recordings of flexible nasolaryngoscopy, or no recording
Outcome	○ Patient satisfaction ○ Patient care ○ Trainee learning ○ Video quality ○ Diagnostic accuracy
Study Design	○ Randomized and non-randomized comparative studies, retrospective and prospective cohort studies, case series ○ Published in English language
Exclusion Criteria	
Population	○ Non-Otolaryngology-trained residents/ physicians
Intervention	○ Endoscopy other than flexible nasolaryngoscopy ○ No use of smartphone adapters
Comparator	○ NA
Outcome	○ No reported outcomes
Study Design	○ Single case reports ○ Case series with *N* < 10 ○ Non-English language

### Data collection and extraction

Data was extracted from the studies using a pre-written data entry form. Titles and abstracts were independently screened by two reviewers (A.Q., L.C) to assess for initial relevance. Titles or abstracts that were deemed relevant by either reviewer were obtained in full document or PDF form. Papers were then screened to determine if they met eligibility criteria, and if so, data was extracted accordingly. Data extraction was completed by two reviewers (A.Q., L.C.) and included important clinical baseline variables as well as primary outcomes measures (Tables 2, 3). All disagreements between reviewers were discussed and resolved by a consensus meeting including all four authors.

**Table 2:** Included study characteristics

Study name	Year	Type of Study	Patients (N)	Exams recorded with endoscope adapter (N)	Exams recorded with endoscopy video tower (N)	Scope operator trainee level	Model of scope adapter	Model of endoscopy video tower	Model of smartphone	Safety/ Privacy measures
Liu H et al..	2016	Prospective cohort	30	30	30	Staff, residents (all levels)	ClearScope (Clearwater Clinical Limited, Ottawa, Canada)	KayPentax (HOYA Corporation, Pentax Lifecare Division, Tokyo, Japan)	iPhone (Apple, Cupertino, CA)	Modica (Clearwater Clinical Limited, Ottawa, Canada)
Liu YF et al	2016	Prospective cohort	43	43	0	Residents (PGY-1, −2)	ClearScope	NA	NA	NA
Lozada et al	2017	Prospective cohort	79	79	0	Residents (PGY-1)	Mobile Optyx (MobileOptyx, Philadelphia, PA)	NA	iPhone	Dedicated team iPhone

**Table 3:** Included study outcomes

Study name	Year	Primary Outcomes of Interest	Secondary Outcomes	Outcome Measures	Findings
Liu H et al	2016	Diagnostic accuracy	NA	Controlled blinded comparison of scope adapters and endoscope tower recorded videos	No significant difference between scope adapter and endoscopy tower videos (mean difference = 1.54%, p = 0.69).
		Video recording quality		5-point Likert scale across 7quality variables	No significant difference across 7 categories (*p* = 0.11–0.92)
Liu YF et al	2016	Resident Education	NA	Resident and attending self-ratings of educational value of scope adapter examinations (non-validated 5-point scale)	Residents felt that reviewing examinations recorded with scope adapters enhanced learning in 79% of cases, and that ability to discuss recorded exams with attendings enhanced learning in 88% of cases. Attendings felt discussing recordings enhanced learning in 81% of cases.
Lozada et al	2017	Diagnostic accuracy	NA	Event rates of discordant diagnoses between staff/ resident based on smartphone adapter recordings; χ^2^ to compare frequency of discordant diagnoses across diagnostic categories	11% frequency of discordant exams; No statistically significant difference in number of discordant diagnoses among diagnostic categories
		Video recording quailty		Event rate of repeated examinations	1.3% of exams needed to be repeated due to poor recording quailty

### Data items

The baseline variables that were extracted from each article included: type of exams performed (scope adapter +/− endoscopy video tower recording), scope operator trainee level, model of scope adapter and (when applicable) endoscopy tower used, model of smartphone used, and safety/ privacy measures. Primary outcomes of interest were: 1) patient care impacts of scope adapter video recordings, 2) resident educational impacts of scope adapter video recordings, 3) diagnostic accuracy of flexible nasolaryngoscopy videos recorded with smartphone adapters, and 4) costs of smartphone adapters for flexible nasolaryngoscopy. Secondary outcomes of interest were: 1) Quality of videos recorded using smartphone adapters compared to endoscopy tower video recordings, and 2) patient satisfaction with the use of smartphone adapters for flexible nasolaryngoscopy.

### Risk of Bias in individual studies

Internal validity of study design and conduct was assessed independently by two reviewers (A.Q., L.C.). For non-randomized studies, the Newcastle-Ottawa Quality Assessment Tool was used [3]. Discrepancies were resolved by a consensus meeting including all four authors.

## Results

**Fig. 1:**
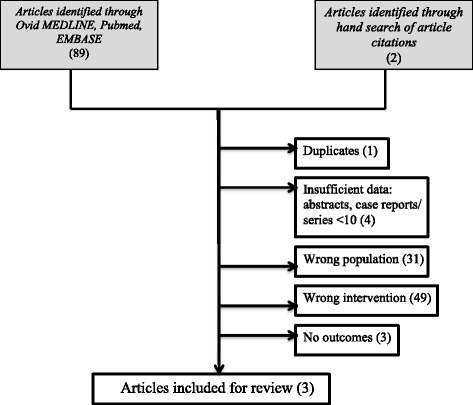
Search strategy and results

A total of 91 studies were screened (Fig. 1). Studies were excluded for: duplicates, different topic/ intervention, non-English language, and insufficient data (abstract only, single case reports, no outcomes data). Three cohort studies were deemed eligible for inclusion [4–6].

### Study characteristics

The total of 152 examinations of patients using smartphone endoscope adapters were reported in the literature. Thirty of these patients also had endoscopy video tower recording of their flexible nasolaryngosocpy exams for direct comparison. The pertinent characteristics of the studies included for review are illustrated in Table 2.

### Study outcomes

Two of the three studies assessed the diagnostic accuracy and quality of videos recorded using smartphone endoscope adapters (Table 3) [4, 6]. In one of these studies (Liu H et al), videos recorded using smartphone endoscope adapters were compared to those recorded using endoscopy video towers, and mean differences in percent correct diagnoses made by blinded observers (senior residents and staff attending physicians) was calculated. The authors found that there was no significant difference in correct diagnoses made between endoscopy video tower recordings and smartphone endoscope adapter recordings (mean difference = 1.54%, *p* = 0.69). This study assessed video quality subjectively using a 5-point Likert scale, with a linear mixed effects model to determine differences in mobile and tower video quality. No significant difference in video quality ratings was found across 7 quality categories (illumination and brightness; ability to identify camera orientation; ability to identify important landmarks/ structures; picture clarify and texture; artefact and background noise; contrast, border, and sharpness; overall satisfaction with video quality) [4]. In the other study (Lozara et al.), videos recorded by postgraduate year-1 residents using smartphone adapters were divided into diagnostic categories (airway evaluation, voice evaluation, dysphagia, aerodigestive tract mass) and were interpreted both by these same residents and staff attending physicians. Chi-squared statistics were used to compare the frequency of discordant exams. The authors found that there was an 11% frequency of discordant exams, with no statistical difference between diagnostic categories. They found that only 1 of 79 (1.3%) of exams had to be repeated due to poor quality [6]. One study (Liu YF et al.) assessed the ability of flexible nasolaryngoscopy videos recorded with smartphone adapters to enhance resident learning. Post-graduate year-1 and -2 residents recorded flexible nasolaryngoscopy exams using smartphone endoscope adapters and then reviewed recorded videos with staff attending physicians, and subsequently were surveyed using a 5-point Likert scale on whether they believed the discussions afforded by the use of smartphone adapters enhanced their learning. The authors found that residents reported that reviewing videos they had recording using adapters enhanced their learning in 79% of cases, and that the ability to discuss video findings with attending physicians enhanced their learning in 88% of cases as reported by attendings, and 81% of cases as reported by residents [5].

Two studies discussed methods employed to protect patient confidentiality. Liu H et al... used a Health Insurance Portability and Accountability Act (HIPAA)- compliant mobile application to record and store images and videos. Lozada et al. used a dedicated team iPhone, with encrypted email and password-secured files and computers. The third study (Liu YF et al) did not comment on the method used to ensure patient privacy and confidentiality.

### Risk of Bias

The Newcastle-Ottawa Quality Assessment tool was used to appraise the selected studies (Additional file 1: Table S1) [3]. The strength of evidence was overall low to moderate quality, with a Newcastle-Ottawa score from 5 to 9 (range 0–9; a lower score indicates methodological weakness). The analysis of the methodologic quality indicated that the principal risks of bias were a lack of objective data comparing outcomes of smartphone endoscope adapter recordings to recordings made with endoscopy video towers, and lack of objective outcome assessment.

## Discussion

Flexible nasolaryngoscopy is an essential tool to the practicing Otolaryngologist. Smartphone endoscope adapters which allow video recording of flexible nasolaryngoscopy examinations are relatively new devices with a number of potential benefits, including enhanced patient care and satisfaction by means of fewer repeat examinations; enhanced resident education by virtue of the ability to store, analyze, and discuss findings of videos recorded in remote locations such as emergency departments and inpatient wards; and decreased costs compared to fixed endoscopy towers. There have been very few studies objectively evaluating the effects of these devices in Otolaryngology practice.

In the present study, we have systematically reviewed the literature and found three studies which assessed the diagnostic accuracy, video quality, and educational benefits of smartphone endoscope adapters. These studies reported heterogeneous outcome data, but overall suggested a benefit of smartphone adapters on resident education, and demonstrated high diagnostic accuracy and video quality with the use of these devices. Lieu et al. [4] objectively compared diagnostic accuracy and quality between videos recorded with endoscopy towers and smartphone adapters and found no difference in either metric. A study of diagnostic accuracy and video quality of smartphone adapters, by way of demonstrating staff physician ability to come to diagnostic and management decisions based on videos recorded with smartphone adapters, identified a low rate of repeat examinations as a result of poor quality (Liu et al., 2016) [6]. Lozada et al. (2017) used self-reported surveys to show a resident educational benefit of smartphone adapters [5]. Outcome data was unable to be combined due to its heterogeneous nature. No study in the literature objectively examined resident educational benefits of smartphone adapters, patient care outcomes with the use of smartphone adapters, patient satisfaction with the use of adapters, or cost-effectiveness smartphone adapters.

**Fig. 2:**
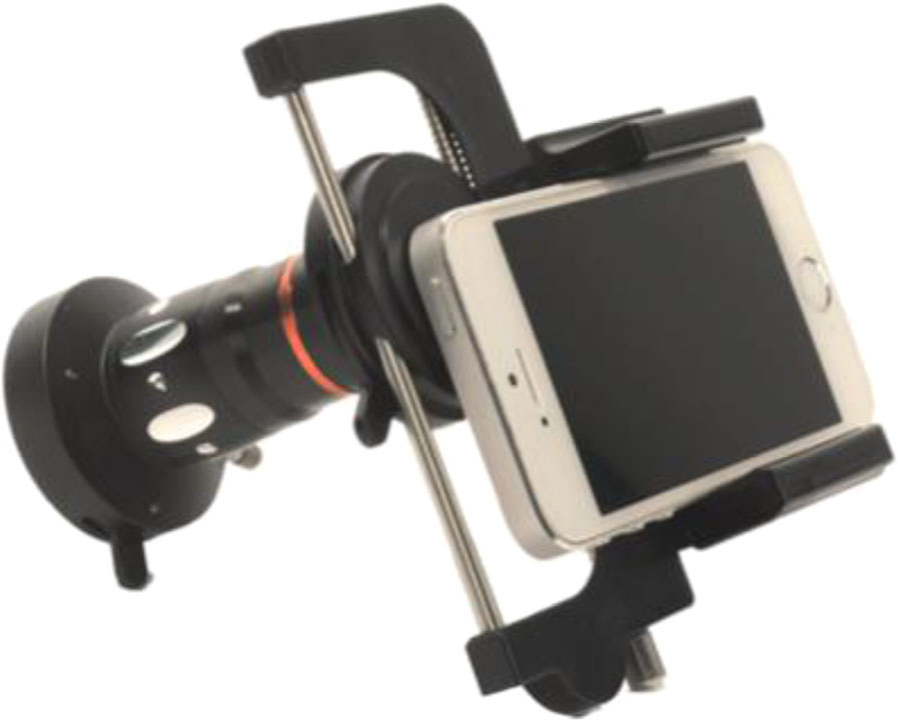
Mobile endoscope adapter (ClearScope; Clearwater Clinical Limited, Ottawa, Canada)

At our own centre (The Ottawa Hospital, Department of Otolaryngology- Head & Neck Surgery), a tertiary care academic centre serving a catchment area of 1.2 million people, residents have been provided with and utilized smartphone endoscope adapters over a five-year period (ClearScope; Clearwater Clinical Limited, Ottawa, Canada) (Fig. 2). Since their introduction, smartphone endoscope adapters have improved cross-departmental communications, being used in grand rounds, interdisciplinary meetings, and teaching rounds. The recordings made using these devices are securely shared with healthcare professionals including members of the OTOHNS team, anesthesiologists, and respiratory therapists, and have improved shared-decision making amongst airway consultants. Furthermore, a database of interesting cases has been curated, proving useful for medical education and research purposes. Endoscopic recordings are included in electronic medical records (EMRs) to ensure improved continuity of care in team handovers. Resident and staff physicians have reported that the frequency of repeat endoscopy by attending physicians to confirm resident diagnoses has decreased, as has the cleaning and maintenance costs associated with using a greater number of flexible scopes.

A variety of models of smartphone endoscope adapter are available on the market. We critically appraised the literature for commercially available smartphone endoscope adapters. There were no head-to-head comparisons of these products available in the published literature. Additional file 2: Table S2 summarizes commercially available devices.

Patient privacy and confidentiality is one concern which has increased in the era of omnipresent smartphone cameras and video recordings [7–9]. Smartphones can be misplaced or hacked, resulting in the breach of private medical information. Furthermore, images captured on smartphones are often stored in insecure mobile applications, many of which automatically sync the image to non-HIPAA-compliant cloud servers such as iCloud, Google+, and Dropbox. Conversely, encrypted mobile applications allow physicians to securely capture and save images and videos; some also provide HIPAA-compliant cloud sync services, allowing physicians to securely backup and share their photos and videos with the rest of the patient’s healthcare team. Additional file 3: Table S3 summarizes available HIPAA-compliant mobile applications for storage of captured images and videos. In our own department, MODICA (Clearwater Clinical Limited; Ottawa, Canada) was formerly used.

There are several important limitations of the present study. There were only a small number of studies published in the literature examining the effects of smartphone endoscope adapters for FNL. Among existing studies, there was a lack of objective data examining our outcomes of interest, including lack of validated surveys; cost-effectiveness analyses; small patient populations; standardization between device operator level of training; and non-uniform use of a variety of different available adapters, endoscope video towers, and smartphones. Two of our three included studies were of poor quality based on Newcastle-Ottawa Scale ratings, due to lack of comparability data within the studies, and lack of objective outcome assessment (Additional file 1: Table S1). As well, among the three included studies, only two types of smartphone adapters were used (ClearScope, Clearwater Clinical Limited, Ottawa, Canada; and Mobile Optyx, MobileOptyx, Philadelphia, PA), and our own departmental experience is also with the ClearScope. The generalizability of our findings – especially scope video quality and diagnostic accuracy – is therefore limited by a lack of data derived from the use of other commercially available scope adapter products (Additional file 2: Table S2). Despite these limitations, we are able to conclude that the present study provides a sufficient overview of the current literature examining the use of smartphone adapters for flexible nasolaryngoscopy. In implementing our search strategy and study design as per the Cochrane handbook and PRISMA guidelines, we were able to effectively appraise the studies meeting our inclusion criteria.

## Conclusion

The market for smartphone endoscope adapters has slowly evolved over the last decade such that new and innovative technology is now available for healthcare professionals to utilize. Accompanying these are a variety of HIPAA-compliant mobile applications to ensure the secure storage and sharing of captured images and videos. Few studies exist examining the utility of smartphone endoscope adapters in OTOHNS practice. This study has systematically reviewed the literature on the use of smartphone endoscope adapters. It has served to identify a significant lack of objective evidence exploring the use, benefits, and cost-effectiveness of these devices. However, we have shown that existing data supports the diagnostic accuracy, video quality, and educational benefits of smartphone endoscope adapters for flexible nasolaryngoscopy. Our study highlights the need for further research into the effects of incorporating these devices into practice.

## Additional files

Additional file 1: **Table S1.** Summary of critical appraisal of included studies using the Newcastle-Ottawa Quality Assessment tool for cohort studies. (DOCX 14 kb)

Additional file 2: **Table S2.** Commercially available smartphone endoscope adapters. (DOCX 124 kb)

Additional file 3: **Table S3.** Commercially available Health Insurance Portability and Accountability Act (HIPAA)-compliant secure mobile applications. (DOCX 46 kb)

## Abbreviations

EMR: Electronic medical record
FNL: Flexible nasolaryngoscopy
HIPAA: Health Insurance Portability and Accountability Act
OTOHNS: Otolaryngology- Head & Neck Surgery
PRISMA: Preferred Reporting Items for Systematic reviews and Meta-analyses
