# Supplementary material for: Smartphone adapters for flexible Nasolaryngoscopy: a systematic review
Source: J Otolaryngol Head Neck Surg. 2018 May 8;47:30. doi: 10.1186/s40463-018-0279-6 (PMC5941791; doi:10.1186/s40463-018-0279-6)
Supplement: Supplementary file 1 — Table S1. Summary of critical appraisal of included studies using the Newcastle-Ottawa Quality Assessment tool for cohort studies. (DOCX 14 kb) [file 40463_2018_279_MOESM1_ESM.docx]

| Authors | Selection | | | | Comparability | Outcome | | | Overall methodologic quality and score (Maximum: 9) |
| --- | --- | --- | --- | --- | --- | --- | --- | --- | --- |
|  | Representativeness of the exposed cohort  (Maximum: ★) | Selection of the non-exposed cohort  (Maximum: ★) | Ascertainment of exposure  (Maximum: ★) | Outcome of interest not present at start of study  (Maximum: ★) | (Maximum: ★★) | Assessment of outcome  (Maximum: ★) | Follow-up long enough  (Maximum: ★) | Adequacy of Follow-up  (Maximum: ★) |  |
| Liu H et al (2016) | ★ | ★ | ★ | ★ | ★★ | ★ | ★ | ★ | Good (9) |
| Liu YF et al (2016) | ★ |  | ★ | ★ |  |  | ★ | ★ | Poor (5) |
| Lozada et al (2017) | ★ |  | ★ | ★ |  |  | ★ | ★ | Poor (5) |
