# Supplementary material for: Smartphone adapters for flexible Nasolaryngoscopy: a systematic review
Source: J Otolaryngol Head Neck Surg. 2018 May 8;47:30. doi: 10.1186/s40463-018-0279-6 (PMC5941791; doi:10.1186/s40463-018-0279-6)
Supplement: Supplementary file 2 — Table S2. Commercially available smartphone endoscope adapters. (DOCX 124 kb) [file 40463_2018_279_MOESM2_ESM.docx]

| **Commercially Available Smartphone Adapters** | **Cost** | **Pros** | **Cons** |
| --- | --- | --- | --- |
| **High Definition Video Endoscopy Tower**  *(Eg: Storz)* | $60,000 CAD | - - High-definition images   - Ability to record and store video | - - Minimally portable   - Difficult to move images off the tower for sharing |
| **Clearwater Clinical ClearSCOPE**  *https://www.modica.md/clearscope/* | $599 CAD | - - Shown in studies to have equivalent image quality to previous gold standard^3^   - Universal adapter – fits all modern smartphones   - Built-in Magnification | - Larger adapter than some alternatives - Can be used on any smartphone device |
| **Endoscope-i**  *http://endoscope-i.com* | 1. GBP | - Very small and portable | - - Not universal across smartphone devices   - No magnification   - Not listed by regulatory agencies such as Health Canada, CE or the FDA |
| **MobileOptx**  *http://mobileoptx.com* | $699 USD | - Quick set up - Built-in magnification | - - Not universal   - Low quality optics |
| **RVA Smart-Clamp**  *https://www.rvasynergies.co.uk* | 99 GBP | - Solid metal design - Universal adapter | - - Not listed by regulatory agencies such as Health Canada, CE or the FDA   - Designed for industry |
| **Storz SMART SCOPE**  *https://www.karlstorz.com/* | $790 USD | - Fits a variety of smartphone models - Built-in magnification - Combined with high-powered light source | - - Not listed by regulatory agencies such as Health Canada, CE or the FDA   - Designed for industry |
