# Supplementary material for: Smartphone adapters for flexible Nasolaryngoscopy: a systematic review
Source: J Otolaryngol Head Neck Surg. 2018 May 8;47:30. doi: 10.1186/s40463-018-0279-6 (PMC5941791; doi:10.1186/s40463-018-0279-6)
Supplement: Supplementary file 3 — Table S3. Commercially available Health Insurance Portability and Accountability Act (HIPAA)-compliant secure mobile applications. (DOCX 46 kb) [file 40463_2018_279_MOESM3_ESM.docx]

| **Commercially Available HIPAA-Compliant**  **Mobile Applications** |
| --- |
| **PicSafe Medi** (PicSafe)  *https://www.karlstorz.com/* |
| **RxPhoto** (AppwoRx)  *http://myappworx.com/rxphoto/* |
| **ShareSmart** (Think Tank Innovations)  *http://sharesmart.ca* |
